# Current evidence of randomized clinical trials on hospice-eligible patients with agitation and dementia: A narrative review

**DOI:** 10.1017/S1478951525101119

**Published:** 2025-11-27

**Authors:** Olivia (YuHsuan) Wang, Noam Calev, Jacobo Mintzer, Arianne Fritts, Doris P. Molina-Henry, Joshua D. Grill, Rema Raman

**Affiliations:** 1 Leonard D. Schaeffer Center for Health Policy & Economics, University of Southern California, Los Angeles, CA, USA; 2 Strategically Focused Research Network: Science of Diversity in Clinical Trials Fellow, American Heart Association, Dallas, TX, USA; 3 Medical University of South Carolina, Charleston, SC, USA; 4 Alzheimer’s Therapeutic Research Institute, University of Southern California, San Diego, CA, USA; 5 Institute for Memory Impairments and Neurological Disorders, University of California, Irvine, CA, USA

**Keywords:** End-of-life, dementia, agitation, randomized controlled trials

## Abstract

**Background:**

Agitation is one of the most distressing behavioral symptoms among hospice-eligible patients with dementia. It compromises patient comfort, increases caregiver burden, and undermines the quality of end-of-life care. Although pharmacologic treatments are frequently used, evidence guiding their safe and effective use in this population remains limited.

**Objective:**

To identify and synthesize existing randomized controlled trials (RCTs) that evaluated interventions for agitation in hospice-eligible patients with dementia, and to assess the quality and relevance of current evidence.

**Methods:**

A narrative review was conducted using systematic search strategies to identify RCTs published between 1990 and 2024 across 8 databases. Studies were included if they (1) focused on hospice-eligible patients with dementia, (2) targeted agitation as a major outcome, and (3) used an RCT design. Studies lacking eligibility criteria, non-RCTs, and non-English articles were excluded.

**Results:**

Of 44 articles screened, only 3 met the inclusion criteria: 2 studies tested nonpharmacological interventions (Namaste and Balancing Arousal Controls Excesses) and 1 tested a pharmacological intervention (sertraline). Results from the nonpharmacological interventions were mixed, and the pharmacologic trial showed no difference between treatment and placebo. Common limitations included small sample sizes, lack of racial and gender diversity, and absence of home-based hospice settings.

**Significance of results:**

There is a critical gap in high-quality and generalizable evidence to guide agitation management at the end of life for patients with dementia. Addressing this gap is essential to improving not only symptom control but also to preserving dignity and reducing caregiver distress in hospice care. Future trials must include diverse populations, incorporate home-based hospice settings, and rigorously evaluate both pharmacologic and nonpharmacologic interventions to support compassionate, patient-centered care.

This review highlights urgent gaps in research and care delivery, underscoring the need for inclusive and scalable intervention designs to address agitation at the end of life.

## Introduction

Agitation is a common and distressing behavioral symptom in patients with advanced dementia, particularly in hospice-eligible populations (Sadowska et al. 2025). It includes increased motor activities, restlessness, aggressiveness, and mental distress. Agitation is commonly measured by the Cohen-Mansfield Agitation Index (CMAI), and higher scores have been associated with poorer quality of death, elevated caregiver burden, increased use of professional caregivers, and greater healthcare costs (Cohen-Mansfield, 1991, 1997; Eisenmann et al. 2020; Gerlach et al. 2023; Jones et al. 2021; Moon et al. 2018; Patrick et al. 2022). Hospice-eligible dementia patients are defined as individuals with advanced dementia and a prognosis of 6 months or less, according to Medicare hospice eligibility criteria or equivalent validated prognostic tools. In the terminal stage, patients may be non-verbal and bed-bound yet still experience significant agitation, affecting up to 71% of this population in the last years of life (Hendriks et al. 2015; Mintzer, 2022).

Despite its prevalence, guidance for agitation management at the end of life remains limited. Approximately 73% of hospice-eligible patients with agitation receive psychotropic medications such as atypical antipsychotics and antidepressants (Kwon et al. 2017), which can cause sedation, confusion, and extrapyramidal symptoms, potentially creating emotional disconnection between patients and caregivers (Marder, 2007; Sadowska et al. 2025). While nonpharmacological interventions and selected pharmacologic agents (e.g., SSRIs) have been tested in advanced dementia, few studies specifically target hospice-eligible populations, where altered pharmacodynamics, polypharmacy risks, and distinct care goals may influence efficacy and tolerability (Broers and Bianchi, 2024; Chen et al. 2023; Cummings et al. 2015; Hermush et al. 2022; Pautex et al. 2022; Porsteinsson et al. 2014; Schneider et al. 2016).

Previous reviews have examined palliative care in dementia broadly but have not synthesized evidence specific to agitation management in hospice-eligible patients (Agar, 2020; Senderovich and Retnasothie, 2020). Given the high prevalence and clinical impact of agitation, a focused review of randomized controlled trials (RCTs) in this group is warranted. This review aims to identify and evaluate RCT evidence for agitation management in hospice-eligible dementia patients, highlight methodological and evidence gaps, and inform priorities for clinical practice and future research.

## Methods

We conducted a structured literature search for published RCTs up to 2024 via MEDLINE, Pubmed, PsycINFO, Proquest, CINAHL, EBSCO, EMBASE, and Cochrane Library. Search terms included combinations of end-of-life, late-stage dementia, agitation, terminal restlessness, terminal delirium, and RCTs.

Studies were eligible if they: (1) included hospice-eligible dementia patients, defined as individuals with advanced dementia and a prognosis of ≤6 months, or a Functional Assessment Staging Tool (FAST) score ≥7a (Luchins et al. 1997; Sclan and Reisberg, 1992); (2) targeted agitation as a primary or secondary outcome; and (3) were RCTs. Hospice eligibility had to be explicitly defined using a hospice setting or a validated prognostic tool. We excluded non-RCT designs, studies without clear hospice eligibility measurement, non-English publications, and unpublished trials.

Of the 44 articles identified, 17 RCTs did not include terminally ill patients with dementia and agitation, 13 did not include agitation as an outcome variable, 6 were not RCTs, 2 were not completed or published trials, 2 were written in German, and 1 could not be retrieved.

## Results

Three RCTs met the inclusion criteria, comprising 2 nonpharmacological and 1 pharmacological intervention (Froggatt et al. 2020; Kovach et al. 2004; Magai et al. 2000). All trials were conducted in residential care settings, and none were conducted in home hospice environments. Table 1 summarizes the study settings, designs, sample characteristics, interventions, and key findings.

**Table 1.** Clinical trials on hospice-eligible patients with dementia and agitation

Authors	Study design	N	Age (years), mean (SD)	Gender (% of females)	Race/Ethnicity, *n* (%)	Inclusion & exclusion criteria; measurement of agitation	Treatment vs. control	Outcome	Results
Table 1-1. Nonpharmacological approach									
Froggatt et al. (2020)^a^	24-week multicenter, placebo-controlled clustered RCT	32	79.0 (10.5)	55.6%	White: 31 (94.4%) African Caribbean: 1 (5.4)	Inclusion: FAST stage 6 or 7, prognosis <3 months Exclusion: Prognosis <1 week, bed-bounded. Measurement: CMAI	Namaste Care (*n* = 18) vs. usual care (*n* = 14)	QoL; quality of death; agitation	QoL improved during intervention; agitation fluctuated and not significantly improved
Kovach et al. (2004)^b^	Multicenter, double-blinded, placebo-controlled RCT	78	86.6 (6.2)	91.0%	Not reported	Inclusion: FAST stage 6 or 7& MMSE Exclusion: Other chronic psychiatric diagnosis; receiving treatment for acute illness Measurement: Visual analog scale	BACE group (*n* = 36) vs. usual care (*n* = 42)	Agitation	Significant reduction in agitation in intervention group (mean score decreased from 39.0 to 30.5); control group unchanged (32.6–32.3)
Table 1-2. Pharmacological approach									
Magai et al. (2000)^b^	8-week, monocentric, double-blinded, placebo-controlled RCT	31	89.2 (6.3)	All female	White: 26 (84%)	Inclusion: Terminal stage of AD with depressive symptoms Exclusion: Patients on medication of antipsychotics or antidepressants; patients with other severe illnesses such as cancer. Measurement of agitation: CMAI	Sertraline (*n* = 17) vs. Placebo (*n* = 14)	Depression (primary); agitation (secondary)	No significant difference in agitation scores between groups; both improved over time

Abbreviation & Note:

FAST: Functional Assessment Staging Tool.

CMAI: Cohen-Mansfield Agitation Inventory.

QoL: Quality of Life.

BACE: Balancing Arousal Controls Excesses.

MMSE: Mini-Mental State Examination.

a. Studies were conducted in the United Kingdom.

b. Studies were conducted in the United States.

### Nonpharmacological approach: Namaste Care (Froggatt et al. 2020)

This UK-based cluster RCT enrolled 32 participants (mean age 79) with dementia and a less-than-three-month prognosis. Participants were predominantly White; only 1 African Caribbean participant was recruited. The intervention consisted of twice-daily sessions emphasizing person-centered activities, meaningful engagement, and sensory comfort to enhance quality of life, and consequently foster emotional connection and reduce behavioral symptoms. While CMAI scores fluctuated, qualitative observations suggested improved affect and interaction during the intervention.

### Nonpharmacological approach: balancing arousal controls excesses (BACE) (Kovach et al. 2004)

This US-based parallel-group RCT recruited 78 participants (mean age 86.6; 91% female) with moderate-to-severe dementia, including hospice-eligible individuals. The intervention scheduled individualized activities to balance high – and low-arousal states. CMAI scores decreased from 39.0 to 30.5 in the intervention group, compared to minimal change in controls (32.6–32.3).

### Pharmacological approach: sertraline trial (Magai et al. 2000)

This US-based double-blind, placebo-controlled randomized trial enrolled 31 female nursing home residents (mean age 89) with late-stage Alzheimer’s disease and co-morbid depression. Medication was administered as follows: Weeks 1–2, 25 mg/day; Weeks 3–4, 50 mg/day; Weeks 5–8, 100 mg/day. Agitation, assessed as a secondary outcome using the CMAI, decreased over time in both groups, but between-group differences were not statistically significant.

## Discussion

Of the studies identified through our systematic search, only 3 met the inclusion criteria, highlighting the scarcity of high-quality evidence for agitation management in hospice-eligible dementia patients. All trials were conducted in residential care settings, and none in home hospice environments, limiting generalizability to community-based care. Reporting of socio-demographic variables was incomplete (see Table 1); for example, Kovach et al. (2004) did not report race or ethnicity, and Froggatt et al. (2020) recruited predominantly White participants (96.9%) with only 1 African Caribbean participant, despite the ethnic diversity of the UK population (Office for National Statistics, 2022). Such gaps restrict the applicability of findings to diverse patient populations.

The nonpharmacological interventions varied in aims and design. BACE, a parallel-group RCT, directly targeted agitation reduction and showed measurable improvement in CMAI scores. Namaste Care, a cluster RCT, prioritized quality of life, with qualitative reports of improved affect and interaction but variable quantitative agitation outcomes. The single pharmacologic trial, a double-blind, placebo-controlled RCT of sertraline, found no significant benefit for agitation compared to placebo. Its all-female sample, depression-focused design, and focus on depressive symptoms rather than agitation as a primary outcome substantially limit the generalizability of findings to broader hospice-eligible dementia populations.

Collectively, these findings underscore the complexity of managing end-of-life agitation in dementia and the methodological challenges of conducting RCTs in this setting. Novel nonpharmacological strategies, such as the Hands of Comfort technique using warm gloves (Julião et al. 2023), and pharmacologic agents with preliminary promise (e.g., dextromethorphan-quinidine, cannabinoid-based compounds) have yet to be evaluated in hospice-eligible populations (Broers and Bianchi, 2024; Cummings et al. 2015; Hermush et al. 2022; Pautex et al. 2022). In sum, only 3 RCTs in the last 34 years met our criteria for inclusion, and no treatments have been evaluated in over a decade. The small evidence base, combined with the limited diversity of study populations and variation in intervention aims, highlights an urgent need for future RCTs to identify, develop, and test both pharmacologic and nonpharmacologic interventions for managing end-of-life agitation in patients with dementia.
